# Automated microfluidic platform for dynamic and combinatorial drug screening of tumor organoids

**DOI:** 10.1038/s41467-020-19058-4

**Published:** 2020-10-19

**Authors:** Brooke Schuster, Michael Junkin, Sara Saheb Kashaf, Isabel Romero-Calvo, Kori Kirby, Jonathan Matthews, Christopher R. Weber, Andrey Rzhetsky, Kevin P. White, Savaş Tay

**Affiliations:** 1 Pritzker School of Molecular Engineering, The University of Chicago, Chicago, IL 60637 USA; 2 Institute for Genomics and Systems Biology, The University of Chicago, Chicago, IL 60637 USA; 3 Department of Chemistry, The University of Chicago, Chicago, IL 60637 USA; 4 Department of Pathology, The University of Chicago Medicine, Chicago, IL 60637 USA; 5 Committee on Genetics, Genomics and Systems Biology, Departments of Medicine and Human Genetics, The University of Chicago, Chicago, IL 60637 USA; 6 Tempus Labs, Chicago, IL 60654 USA

**Keywords:** Lab-on-a-chip, Stem-cell biotechnology

## Abstract

Three-dimensional (3D) cell culture technologies, such as organoids, are physiologically relevant models for basic and clinical applications. Automated microfluidics offers advantages in high-throughput and precision analysis of cells but is not yet compatible with organoids. Here, we present an automated, high-throughput, microfluidic 3D organoid culture and analysis system to facilitate preclinical research and personalized therapies. Our system provides combinatorial and dynamic drug treatments to hundreds of cultures and enables real-time analysis of organoids. We validate our system by performing individual, combinatorial, and sequential drug screens on human-derived pancreatic tumor organoids. We observe significant differences in the response of individual patient-based organoids to drug treatments and find that temporally-modified drug treatments can be more effective than constant-dose monotherapy or combination therapy in vitro. This integrated platform advances organoids models to screen and mirror real patient treatment courses with potential to facilitate treatment decisions for personalized therapy.

## Abstract (web summary)

The use of organoids in personalized medicine is promising but high throughput platforms are needed. Here the authors develop an automated, high-throughput, microfluidic 3D organoid culture system that allows combinatorial and dynamic drug treatments and real-time analysis of organoids.

## Introduction

Cell culture techniques are important tools in both basic and clinical research ranging from personalized or regenerative medicine to more fundamental research like developmental biology. The need for more biologically relevant tissue models has driven interest away from traditional two-dimensional platforms and towards three-dimensional cell culture systems that more accurately simulate cell and tissue morphology, proliferation, differentiation, and migration^1–3^. 3D culture and organoid based systems have been widely used for the study of different disease states, personalized drug screening, discovery drug safety and efficacy studies, and manipulations of cellular environment, ultimately providing more physiologically relevant information and more predictive data for in vivo tests than traditional methods^4,5^.

Patient-derived organoids have several advantages as personalized tumor models. Primary cancer tumor cells cultured as 2D monolayers do not reflect the heterogeneity of the primary tumor due to selection in culture, tissue-specific architecture, and mechanical stresses, while tumor organoids can overcome these deficiencies^6–8^. Patient-derived cancer tumor organoids can be established in a shorter period and are much more economical than costly patient-derived xenograft (PDX) models, which requires a large tissue sample, up to 6 months to establish tumor growth, and retains complications from infiltrating murine stromal cells^9,10^. Tumor organoids can also be cryopreserved, expanded, genotyped, and challenged with therapies within weeks of the initial culture. Additionally, tumor organoids can be cultured from a routine cancer biopsy, such as an endoscopic ultrasound-guided fine needle aspiration, with a high success rate, making it ideal for probing changes involved at different stages of tumorigenesis. Patient-derived organoids also open the possibility of clinical benefits when the response to therapy mimics the parent tumor and allow for deep genomic characterization and ex vivo therapeutic testing in classes of patients that have traditionally been understudied in research settings^9^.

It is desirable to improve the speed, cost, and reproducibility of culture and comprehensive screening of organoids for phenotypes such as drug sensitivity or tissue development. One way to achieve these objectives involves the creation of standardized, parallelized, and miniaturized assays that can be performed in a highly reproducible environment with the utilization of minimal amounts of reagents, using an automated system that allows users to culture and screen thousands of conditions with minimal training and investment. Microfluidics can provide dynamical screens with drug cocktails and signaling molecules, where the concentration, timing, and duration of fluidic delivery can be precisely controlled in an automated fashion. Similar systems have already been developed and commercialized for 2D cultures, but fail to accommodate many 3D cell culture structures due to several limitations of existing microfluidic systems^11–13^. Most importantly, organoids and other 3D cell structure models often require the use of an extracellular matrix that interacts with the cells and tissue to provide both mechanical support and biochemical cues. Naturally derived matrices (e.g., Matrigel) are widely used; however, their physical and chemical properties (such as temperature sensitivity and clogging of microfluidic channels) make current microfluidic and other high-throughput techniques obsolete.

Current microfluidic literature has demonstrated the use of organoids with microfluidics, but either contains very low throughput methods (less than eleven chambers per device), incompatibility with Matrigel, little to no automation, and/or with small chamber depths that are unable to accommodate the large 3D organoid size (∼400 μm diameter)^5,14,15^. Other microfluidic-related devices called organ-on-a-chip or body-on-a-chip platform have used tissue-specific cells and their extracellular matrixes to remodel 3D tissues architectures and physiological conditions, such as shear stress and fluidic flow, within a tissue-specific microfluidic structure and system^16–18^. However, while extremely useful for certain research studies, in comparison to organoid based microfluidics, these systems are limited when reconstituting the biological complexity of tissue development. Also, body-on-a-chip systems and other commercially available automatic and high-throughput methods often required complex or extremely expensive robotic based systems, have complications or incompatible with gel scaffolds, and not always suitable for real-time monitoring of cellular and molecular features^19,20^.

Finally, some of the most common and effective chemotherapies are administered in a specified temporal sequence^21–23^, however culture devices that can accommodate 3D gel-based cultures are typically not automated and do not support the on-demand perfusion of drugs and signaling factors that could provide preclinical drug screens in a time-dependent manner. Furthermore, time-dependent analysis of 3D organoids is extremely challenging, especially when the fluidic conditions need to be dynamically altered during experiments. To address these limitations, we have developed a robust and streamlined automated microfluidic platform that allows high-throughput culture, stimulation, assaying, and harvesting of organoids and other 3D culture models under dynamic conditions. Our system is compatible with gel-based culture and dramatically decreases the labor-intensive and time-consuming tasks of 3D cellular culture, human error, and minimizes the consumption of expensive reagents, while being able to continuously monitor the cultures for long periods of time. Most importantly, the automated fluidic architecture built into the system facilitates dynamic programmed changes to the culture conditions and enables real-time screening of different sequences of drugs or signaling factors in parallel culture chambers. The dynamical control of fluidic conditions allows testing of thousands of drug stimulations in a single experiment. Once an experiment is completed, the cultures can be easily harvested for additional genomic analysis, expansion, or grafting. Ultimately, this system can increase and accelerate the use of organoids and other 3D culture-based systems (i.e., spheroids or cellular aggregates), enhancing their ability to become an essential tool for both basic and translational research.

## Results

### Design of an automated platform for 3D cellular cultures

We developed an automated high-throughput microfluidic platform capable of culturing organoids and other 3D cellular cultures for continuous monitoring of 3D growth, morphology, and biochemical analysis. The platform consists of two integrated devices, a 3D culture chamber device and a multiplexer fluid control device, custom software for automated and programmable experimental control, and live-cell time-lapse fluorescence microscopy.

**Fig. 1:**
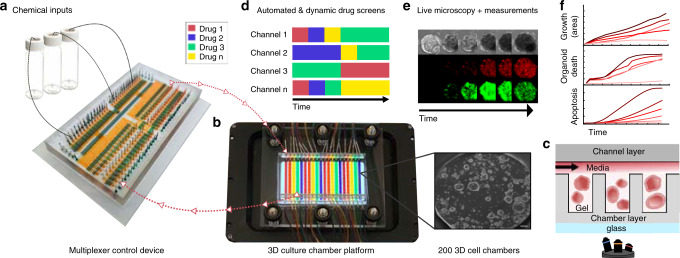
Automated microfluidic 3D cellular and organoid culture platform for dynamical drug perturbations. **a** A programmable membrane-valve-based microfluidic chip (multiplexer control device) provides automated stimulation profiles to various chambers of a separate 3D culture platform (**b**) to produce many parallel and dynamical culture experiments. **b**, **c** The 3D culture chamber platform contains 200 individual chambers that are compatible with temperature-sensitive gels (i.e., Matrigel), and an overlaying channel layer enables 20 independent fluidic conditions (scale bar 100 μm). The channel layer is reversibly clamped on top of the chamber layer to provide media and other chemical stimulation without leakage. **c** A cross-section of the two-layer multichambered PDMS-based 3D culture chamber device. **d** 30 chemical inputs and 30 outlets of the multiplexer control device (**a**) are preprogrammed to provide combinatorial and time-varying stimulations to the 3D culture chamber device (**b**). **e**, **f** Organoids or 3D cellular structures are continuously observed through time-lapse imaging for quantification; fluidic culture conditions can be changed on demand. The 3D culture chamber device can also be disassembled for cell harvesting and further cellular assays.

The reversibly clamped two-layer chamber chip consists of a 200-well array and an overlying layer of fluidic channels. Each well unit in the lower layer serves as a culture for organoids or other 3D cellular structures grown inside a gel-like extracellular matrix such as Matrigel or hydrogel (Fig. 1). A second channel layer compliments the chamber array and is reversibly bonded together, along with a glass slide, through a clamping-based system to provide fluidic channels. The array is divided into 20 different subsets of 10 individual chamber units to accommodate up to 10 different patient samples (Fig. 1b–c, Supplementary Fig. 1).

The 3D culture chamber platform was geometrically engineered to reduce bubble formation and prevent leakage between channels (Supplementary Fig. 2, Supplementary Movie 1). Uniquely, the channel and chamber heights were specifically engineered to provide a suitable 3D environment for the large mature organoids that average around 500 μm in diameter. The fluidic channels are 455 μm in height in order to provide enough liquid nutrients to the growing organoids and to prevent disruption of the gel-based environment in the chambers. The chamber units are 610 μm in height, on average, which is significantly larger than most microfluidic devices (typically between 100–200 μm, although heights of up to 350 μm have been reported^14^). Additionally, previously published high-throughput microfluidic devices are not compatible with temperature-sensitive Matrigel, which quickly solidifies at room temperature and difficult to flow through microfluidic channels and valves. Our two-part, valve-less, non-permanently bonded organoid culture device allows for easy accommodation of Matrigel into the wells through manual pipetting and the clamping feature allows for reversible bonding without the possibility of any leakage of the device after the cells are added, eliminating the need for a permanent bonding method or complicated/unfeasible Matrigel loading methods.

The variable fluidic conditions are supplied to the well chambers via channels that pass over the top of the chambers. This configuration creates 20 independent experimental conditions that are controlled with a second multiplexer device. To formulate automated, complex, and dynamic fluidic flow to the system, we designed a valve-based, reusable multiplexer control device composed of a system of fluidic channels and valves to provide culture control to the valve-less 3D culture chamber device (Fig. 1a, Supplementary Fig. 3). The multiplexer device is automated and controlled by solenoid valves and custom software to carry out preprogrammed experiments and deliver precise fluid sequences to the culture device. The experimental conditions supplied to organoids consist of specific temporal profiles of chemical inputs (i.e., medium, drug cocktails, chemical signals) that are prepared and connected through fluid vials with their delivery preprogrammed through a simple tab-delimited text file (e.g., Excel). The desired solutions are preloaded (up to 30 solutions) to the multiplexer device, which in return provides the automation by acting as the fluidic guide to provide each desire solution to the specific designated channel in a time specific manner. The level of automation allows easy programing and application of any number of dynamic conditions and overcomes the limits of manual pipetting by limiting errors and standardizing timing of media delivery. While the 3D cellular cultures are being exposed to predetermined experimental regimes, they are simultaneously imaged in 3D via phase contrast and fluorescence deconvolution microscopy to provide real-time measurements of cell reactions, movements, and proliferations (Fig. 1d–f). The programmable microscope is also equipped with an environmental chamber (incubator) for continuous temperature and climate control. Once an experiment is completed, the design of the 3D culture chamber device allows the upper fluidic supply channels to be removed exposing the well array with cell-containing gel for facile harvesting of 3D cultures/organoids for subsequent analysis (sequencing, expansion, etc.). Moreover, during the entire culture period, we can use fluorescent cellular protein markers for continuous time-dependent analysis (Fig. 1e, f).

### Individual cells develop into organoids on the platform

**Fig. 2:**
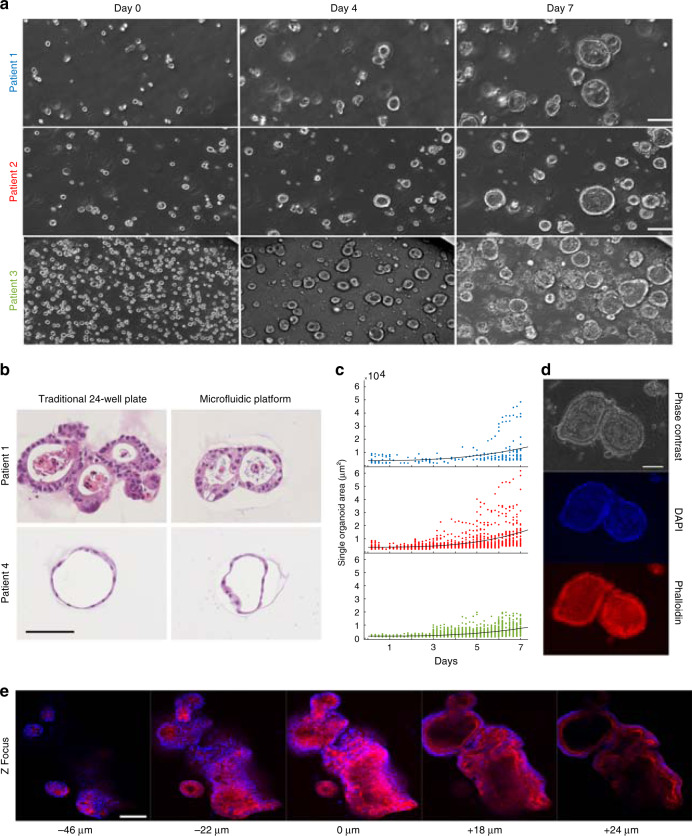
Human tumor organoid culture and growth on microfluidic platform. **a** On-platform growth of organoids: three separate patient-derived pancreatic ductal adenocarcinoma (PDAC) organoids in Matrigel from single cells to formation of differentiated morphology of complex 3D structures (scale bar 100 μm). **b** Organoids from two patients were grown in parallel in a traditional 24-well plate and on our microfluidic platform (scale bar 100 μm). After mature organoid formation, organoids were harvested, H&E stained, and their morphologies compared and analyzed. In both platforms, organoids from patient 1 exhibited back-to-back glands with a high degree of nuclear atypia and pleomorphism with an accumulation of apoptotic luminal necrotic cells. Organoids from patient 4 demonstrated a well-differentiated morphology with simple spherical organoids and uniform nuclear and cytoplasmic features with little or no accumulation of necrotic luminal cells. **c** Organoid growth curves of PDAC organoid samples derived from three patients grown from single cells for 7 days on the platform. Each dot represents the cross-sectional area of an individual organoid. Patient 1 (blue), Patient 2 (red), Patient 3 (green). **d** Long-term culture, growth, and fluorescent staining of fixed PDAC organoids on the platform. Nuclei staining (DAPI) and F-actin (Phalloidin) are demonstrated (scale bar 100 μm). **e** Multiple *Z* image slices or stacks of a group of fixed and fluorescently stained organoids with DAPI and phalloidin (scale bar 100 μm).

Our 3D culture platform has been used to grow a variety of 3D cell structures from a cancer cell line (MDA-MB-231) grown into aggregates, pancreatic tumor organoids from patient-derived samples, and colon organoids from human-derived normal (i.e., non-diseased) colon tissue samples (Fig. 2a,c, Supplementary Fig. 4, Supplementary Movies 4–8). The application of the same platform to grow different 3D models provides an easy means of standardization and allows culturing of dynamic conditions for long time periods (14+ days). For example, pancreatic ductal adenocarcinoma (PDAC) organoids from patient-derived samples are grown from single cells to a stage where tissue-level structures are observable. During the entire culturing period, we conducted continuous visual monitoring of cells and stained cellular components on the platform (Fig. 2d). Timing and feeding were optimized using the high-throughput features of the system to ensure a suitable environment where the cells could proliferate and ultimately grow into 3D glandular structures.

The cellular phenotypes of PDAC organoids grown on our platform were then compared to traditional plate-based experiments and other known tumor tissue characteristics for these same organoids in previously published work^8^ (Fig. 2b). To validate the tumor pathology, a gastrointestinal pathologist compared the architecture and cell morphology of two organoids with very different histological appearances grown on both platforms and then stained with hematoxylin and eosin (H&E) for histopathological evaluation There was a remarkably good correlation between the organoids grown on different platforms for both patients based on the H&E cell appearance and glandular structure of the tumor organoids. The formation of multilumen structures with a high degree of cytologic atypia seen in patient 1 in contrast to the well-differentiated cysts in patient 4 were consistent between platforms. The contrasting patient morphologies are a reflection of the organoids ability to reflect the individualized characteristics of the tumor they are derived from, which is retained between the platforms^8^. To accommodate the entire 3D structure, we took multiple *Z* image slices or stacks to fully image the entire volume of each organoid (Fig. 2e, Supplementary Movies 2–3). Selected *Z* stacks with the largest diameter of the organoid were analyzed by measurements accumulated through image segmentation. The cross-sectional area of multiple individual PDAC organoids was measured over the course of 7 days for three different patients to obtain growth rates (Fig. 2a, c).

### Growth and drug screening of primary human tumor organoids

Once the culture of organoids was established on our platform, we developed a robust high-throughput assay of organoid growth and cellular apoptosis for drug-treated and untreated samples to demonstrate the experimental and potential biomedical utility of our platform. We used FDA-approved and standard of care chemotherapy regimens for pancreatic cancer to design drug screening assays on human-derived pancreatic cancer organoids using our platform. A set of fluorescent dyes were used to monitor the drug sensitivity of each organoid continuously using live-cell imaging. Our design accommodates up to 20 different regimens and 10 different patient samples to be tested in parallel.

**Fig. 3:**
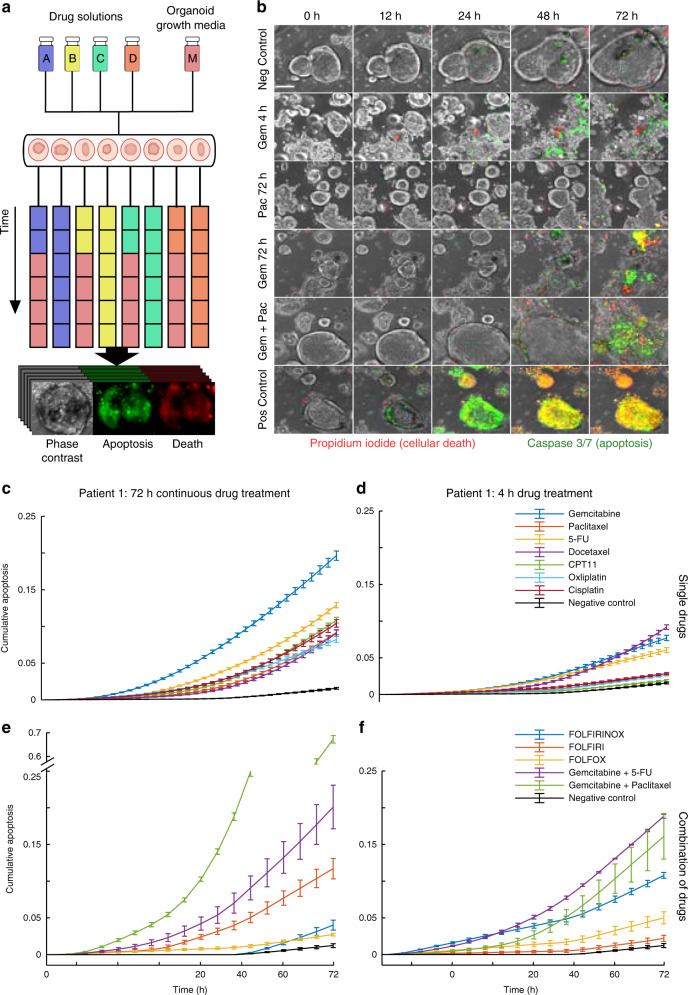
Combinatorial drug treatment of human tumor organoids on microfluidic platform. **a** On-platform drug treatment and stimulation with continuous fluorescence and phase imaging of organoids for the treatment duration. Each color represents a different drug formulation. Drug treatments on each channel can be changed on demand, creating time-varying drug treatments. Organoids can be analyzed for growth, morphology changes, or death. **b** Representative images (10×) of gemcitabine (100 nM) treated organoids for a 4-h drug pulse followed by normal growth media, continuous treatment of paclitaxel (10 nM) for 72-h, continuous treatment of gemcitabine (100 nM) for 72-h, a combination dose of gemcitabine (100 nM) + paclitaxel (10 nM) for 72-h, and negative and positive controls (staurosporine 10 mM). Caspase 3/7 reagent (green) used for apoptosis detection and propidium iodide (red) for dead cells along with phase contrast images (scale bar 100 μm). **c** Average caspase 3/7 signal over 72-h period of continuous single drug treatments for patient 1. **d** Average caspase 3/7 signal over 72-h period for a 4-h pulse of a single drug treatment followed by normal growth media for patient 1. **e, f** 72-h (**e**) and 4-h (**f**) drug treatments similarly examined for multiple known combinations of drugs. **c**–**f** All data presented as mean values ± SEM, *n* = 3, and normalized to positive control. Overall, combination chemotherapy treatment resulted in significantly increased apoptosis in tumor organoids compared to single drug treatments as expected. Source data for panels **e**, **f** are available.

To verify the drug screening capabilities of the platform, we treated PDAC organoids, obtained from three different patients, with clinically relevant doses of gemcitabine (100 nM), paclitaxel (10 nM), 5-fluorouracil (5-FU, 100 nM), docetaxel (10 nM), irinotecan (CPT-11) (100 nM), oxaliplatin (100 nM), and cisplatin (100 nM). The PDAC organoids were grown on the platform for a minimum of 7 days prior to drug exposure or until organoids were visibly mature. We quantified the organoid growth and reactions to drug exposure continuously through automated image analysis using apoptosis and cellular death fluorescent dyes (Fig. 3a–b, Supplementary Movie 9). During the entire course of organoid culture and treatment, image stacks taken of the chamber arrays provided real-time 3D monitoring of organoid size, number, and morphology. The monitoring continued during drug exposure and images were automatically analyzed in combination with fluorescent markers for size and viability using MATLAB based image analysis (Fig. 3c–f). To mimic previously used plate-based methods, the organoids were continuously exposed to individual drugs for 72 h (Fig. 3c, e) or exposed for a 4 h drug/s pulse (Fig. 3d, f) followed by a wash and replacement with normal growth media for the duration of the treatment. At the 48-h mark, all treatment and control groups were supplied with either drug-containing or normal growth media to replenish growth nutrients and prevent non-drug related cell death. We also compared clinically relevant combinations of the chemotherapy drugs administered for 4 or 72 h (Fig. 3e–f, Table 1). This screen allowed for multiple conditions to be automatically supplied to different subsets of organoids, providing an easy means to simultaneously compare multiple treatments in a single experiment. Overall, we found that combination chemotherapy treatment resulted in significantly increased apoptosis in tumor organoids compared to monotherapy as expected.

**Table 1:** Temporal drug combinations.

Combinatorial chemotherapy	Constant drug combination description	Temporal drug combination description	Temporal delivery description
FOLFIRINOX	CPT-11 (100 nM), Oxaliplatin (100 nM), Fluorouracil (100 nM)	CPT-11 (100 nM), Oxaliplatin (100 nM), High Dose Fluorouracil (1 μM), Low-Dose Fluorouracil (100 nM)	CPT-11 (2 h), Oxaliplatin (2 h), High Dose Fluorouracil (30 min), Low-Dose Fluorouracil (48 h)
FOLFIRI	CPT-11 (100 nM), Fluorouracil (100 nM)	CPT-11 (100 nM), High Dose Fluorouracil (1 μM), Low-Dose Fluorouracil (100 nM)	CPT-11 (4 h), High Dose Fluorouracil (30 min), Low-Dose Fluorouracil (48 h)
FOLFOX	Oxaliplatin (100 nM), Fluorouracil (100 nM)	Oxaliplatin (100 nM), High Dose Fluorouracil (1 μM), Low-Dose Fluorouracil (100 nM)	Oxaliplatin (4 h), High Dose Fluorouracil (30 min), Low-Dose Fluorouracil (48 h)
Gemcitabine and Fluorouracil (5-FU)	Gemcitabine (100 nM), Fluorouracil (100 nM)	Gemcitabine (100 nM), Fluorouracil (100 nM)	Gemcitabine (4 h), Low-Dose Fluorouracil (48 h) repeated twice
Gemcitabine and Paclitaxel	Gemcitabine (100 nM), Paclitaxel (10 nM)	Gemcitabine (100 nM), Paclitaxel (10 nM)	Gemcitabine (4 h), Paclitaxel (4 h), normal growth media (24 h) repeated twice

### Dynamic drug screening of human cancer organoids

Some of the most common and effective combination chemotherapies for cancer are clinically administered to the patient in a specified temporal order^21–23^. Our platform can automatically create such dynamic chemotherapy regimens in many parallelized organoid cultures and analyze organoid response in real time. To investigate the efficacy of such treatments, we leveraged several standard medical chemotherapy practices for PDAC to design five temporal chemotherapy regimens. With our platform, we exposed organoids to these regimens to mirror real treatments given to PDAC patients in the clinic^18–21^. Unlike traditional plate-based methods, our platform allows for combination chemotherapy to be sequentially delivered in pulses to the desired array of organoids without human intervention, drastically reducing laborious pipetting steps and human error while maintaining a real-time organoid imaging process.

**Fig. 4:**
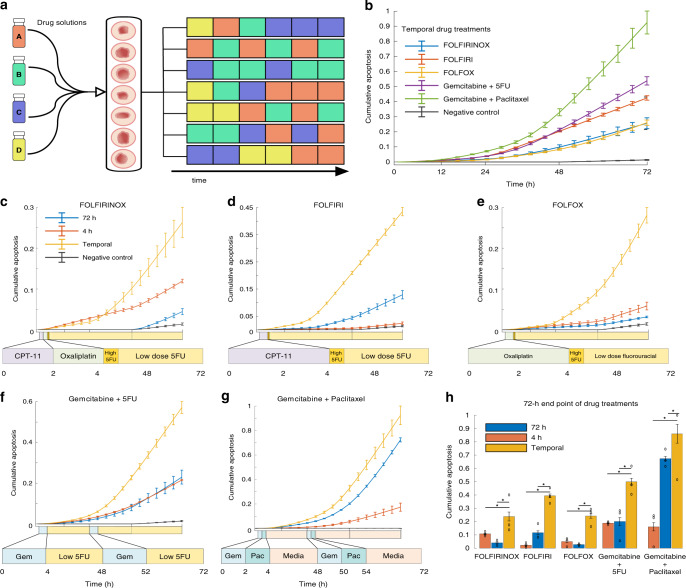
Sequential and temporal drug treatment on the microfluidic platform reveals the efficacy of dynamic temporal drug treatment for personalized therapy. **a** Schematic of sequential drug delivery schedules of single drugs delivered temporally in pulses to recapitulate dynamic combination chemotherapy with the platform. Colors in each row represent a different drug formulation, which can be changed on-demand. **b**–**h** Comparison of the temporal delivery for five combination chemotherapies using average caspase 3/7 signal to detect apoptosis for patient 1. All data presented as mean values ± SEM, *n* = 3, and normalized to positive control. **c**–**g** Comparison of temporal delivery for each of the five combination chemotherapies to their 72-h and 4-h constant delivery counterparts (i.e., all drugs in the sequence at once). Details of the drugs used in each therapy regimen are shown below the graph and described in more detail in Table 1. Time course of each drug on the *x*-axis is to scale. **h** Comparison of all investigated therapies at the end of the 72-h drug treatment period (asterisk denotes significant differences from temporal treatment, two-way ANOVA, *p* values from left to right; FOLFIRINOX: 8.3E-07, 7E-07; FOLFIRI: 1.7E-06, 7E-07; FOLFOX: 0.03, 0.01; Gem+5-FU: 3.9E-05, 8.3E-05; Gem+Pac: 9.6E-10, 6.16E-06). Sequentially-administered combination therapy is more efficient in inducing tumor cell death. Source data for panels **c**–**g** are available.

In the clinic, FOLFIRINOX is a frequently used combination chemotherapy regimen that consists of two hours of intravenous irinotecan (CPT-11), followed by two hours of oxaliplatin, continued with a high dose burst of 5-fluorouracil (5-FU, 1 μM), and finished with a continuous infusion of low-dose 5-FU (100 nM) for 46 h. Our platform mirrored this clinical treatment strategy by exposing the organoids to 2 h of CPT-11, 2 h of oxaliplatin, 30 min of a high dose of 5-FU, and then a continuous low dose of 5-FU until the end of the 72-h experiment. We then compared the temporally and sequentially delivered FOLFIRINOX treatment to a static FOLFIRINOX treatment, where the organoids received all of the complete FOLFIRINOX cocktail at once and continuously for 4 or 72 h, mimicking the capabilities of traditional plate experiments. This process was repeated for four more common combination therapies: FOLFIRI, FOLFOX, gemcitabine + 5-FU, and gemcitabine + paclitaxel as outlined in Table 1 and illustrated in Fig. 4.

### Effectivity of temporally modified drug delivery

Analysis of combinatorial and dynamic drug treatment experiments revealed notable differences between constant and temporally pulsed drug treatments for certain patients. For patient 1, temporally modified delivery of FOLFIRINOX, FOLFIRI, FOLFOX, gemcitabine + 5-FU, and gemcitabine + paclitaxel were the only treatments that produced significantly different responses from the control groups (repeated measurement ANOVA *p* values were, respectively, 0.0098, 8.39E-07, 0.02, 7.05E-07, 2.31E-05). Patient 1 also demonstrated significantly greater drug sensitivity (i.e., increased apoptosis) to almost all of the sequentially-administered combination therapies compared to their 4- and 72-h simultaneous administration counterparts (Fig. 4h). Overall, the gemcitabine/paclitaxel treatment sequence was the most effective on the organoids derived from patient 1. Retrospective clinical data reports that patient was treated with a Whipple procedure, followed by one round of adjuvant gemcitabine after surgery (Table 2). A final round of gemcitabine + paclitaxel was given, but the patient was transferred to hospice shortly after.

**Table 2:** Retrospective clinical data compared to corresponding patient′s organoid drug sensitivity results.

	Clinical results	Organoid results
Patient 1	Whipple procedure with an adjuvant round of gemcitabine. Liver metastases emerged and a round of gemcitabine + paclitaxel (otherwise known as gemcitabine + abraxane in clinic) was given right before the patient expired. Total duration was 11 months.	Organoids were most sensitive to a treatment of gemcitabine + paclitaxel
Patient 2	Whipple procedure followed by delayed adjuvant round of gemcitabine due to surgical complications. Liver metastases emerged and FOLFIRINOX with dose reduction was administered, before the patient expired. Gemcitabine + paclitaxel was planned, but never administered. Total duration was 16 months.	Organoids were most sensitive to gemcitabine and gemcitabine + 5-FU.
Patient 3	Patient received neoadjuvant FOLFIRINOX before the Whipple procedure, followed by an adjuvant round of gemcitabine. Local recurrence and liver metastases emerged. A round of gemcitabine + paclitaxel was administered, but was discontinued due to intolerance before the patient expired. Total duration was 10 months.	Organoids were most sensitive to all gemcitabine-containing therapies

**Fig. 5:**
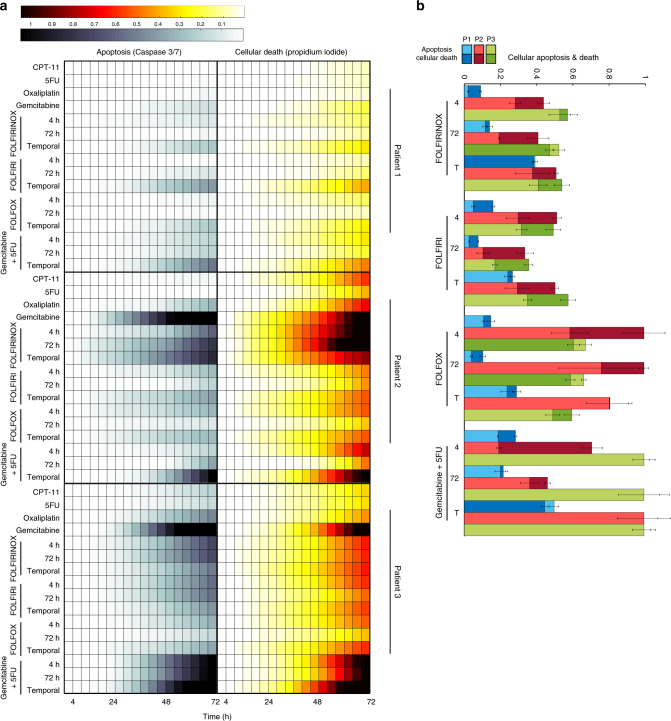
High-throughput drug testing of multiple patients on chip. **a** Heatmaps of organoids from three patients grown and stimulated in a simultaneous experiment. Cellular apoptosis (caspase 3/7) and death (propidium iodide) were recorded over the 72-h drug treatment period to assess drug sensitivity. **b** End-point analysis of average organoid cellular apoptosis and death for each patient and combination treatment group. All data presented as mean values ± SEM, *n* = 3, and normalized to positive control. Response comparisons between the different patients revealed distinct sensitivities to specific drug regimens across both independent cell viability assays. Source data for panels **a**, **b** are available.

The capacity to conduct experiments in parallel allowed us to compare three different patients simultaneously without human intervention (Fig. 5). Patient 2 showed significant drug sensitivity to a 72-h treatment of gemcitabine, FOLFIRINOX delivered both temporally and for 72 h, and temporally-delivered gemcitabine + 5-FU compared to the control (*p* values = , respectively, 7E-07, 9.61E-05, 3.04E-06, 0.01). Patient 2 was the most sensitive to the 72-h gemcitabine and temporally-delivered gemcitabine + 5-FU. This is consistent with previously published organoid sensitivity data^8^ using traditional plate-based testing methods. In the clinic, patient 2 received adjuvant rounds of gemcitabine followed by FOLFIRINOX with dose reduction before ultimately being transferred into hospice. It was also noted that the patient was delayed in receiving chemotherapies due to surgical complications. Gemcitabine + paclitaxel was also planned, but never administered due to fast progression of the disease.

Revealing more additional patient-specific drug sensitivity, patient 3 showed all gemcitabine-based treatments and durations significantly differ from the control (Ctrl vs. Gem *p* = 8.39E-07; Ctrl vs. Gem + 5-FU 4 hr *p* = 6.72E-05, 72 hr *p* = 0.0004, Temporal *p* = 1.65E-06). In clinic, patient 3 was administered a round of neoadjuvant FOLFIRINOX, followed by a round of adjuvant gemcitabine after the Whipple procedure, but the tumor ultimately metastasized to the liver. A round of gemcitabine + paclitaxel was administered, but discontinued due to patient′s intolerance and quick decline in health. Overall, these results demonstrate the importance of studying personalized drug responses, as individual patients exhibit different responses to drug treatment regimens.

In order to assess reproducibility and the effect of passage number, we repeated this experiment with patients 1 and 3 (Fig. S5). Two different passage numbers (41 and 15) for patient 1 were compared and still revealed the most significant sensitivity to temporally derived gemcitabine + 5-FU over the 72- and 4-h durations in both experiments. Similar passage numbers (24 and 27) for patient 3 were also compared in two different experiments; again, both passages showed similar significant drug sensitivities for all four gemcitabine treatment groups, with temporal delivery as the most effective. The consistent response across passages highlights the reproducibility of the platform.

## Discussion

Here, we have developed an automated organoid culture platform for dynamic and combinatorial drug screening to aid in the research and modeling of human organ development and human pathologies. Our microfluidic platform enables highly reproducible, dynamic, and robust experimental analyses of organoids, while also accommodating the use of complex treatment combinations and temporal sequences of culture conditions. Traditional 2D immortalized or primary monolayer cell lines do not reflect the heterogeneous structure of in vivo tissue, while recently developed approaches for culturing 3D models, such as organoids, can overcome these limitations. The many potential applications of these new models are only just beginning to be explored and the described platform is a means to accelerate the use of organoids and other 3D cellular structures in fundamental and preclinical research.

As demonstrated here, the automated, high-throughput, and dynamic capabilities of the platform allow simultaneous parallel comparison between organoids grown from multiple patients under a variety of individual and combination drug therapies, as well as using predetermined temporal sequences of drug treatments. This design allows robust determination of drug effects in three ways. First, the complex addition of reagents in precise amounts at precise times would be a major source of error if attempted manually. Using our microfluidic architecture, volumes are simply metered by the number of pump cycles or level of pressurized flow and the device geometry. Reagent additions (e.g., drug exposure times, number of drug pulses, drug combinations) are programmed into the control system, that is error free as it is specified in a control file and then translated into the fluidic control architecture, thus the accuracy depends only upon correct connection of supply fluid vials.

The second way in which this experimental platform provides robustness is via the large number of repeated conditions and the intrinsic inclusion of controls through identical well units exposed to identical conditions. This strategy provides the necessary data to obtain statistically relevant quantifications of experimental outcomes. Finally, the temporal capabilities of the platform to deliver drugs individually in a sequential manner enables testing thousands of drug combinations to procedurally mirror real-life patient treatments.

Our results showed significant differences in the drug response of individual patients, highlighting the importance of studying drug treatments at the individual level, particularly in cancer. We also found that temporal drug delivery may be more effective for certain patients than continuous or single-pulsed simultaneous drug treatment administered during organoid screening. This temporal effect warrants continued exploration and could lead to clinical trial hypothesis by testing a larger number of patient organoids, different type of cancer organoids, and testing different sequence variations of standard of care drug combinations.

In comparison to the clinical retrospective data, patient 1′s organoids were most sensitive to gemcitabine + paclitaxel and unfortunately this treatment was delivered late in the treatment regimen as the last chemotherapy administered to the patient. This was also at a point of advance progression of the disease, where as soon as the chemotherapy administration was completed, the patient was transferred to hospice. Given the organoids’ sensitivity results, the patient could have potentially benefited by receiving the chemotherapies earlier in the treatment plan. Patient 2 received gemcitabine and FOLFIRINOX postsurgery before being transferred to hospice. Gemcitabine and gemcitabine + 5-FU were the most effective with the organoids and the patient could have benefited from receiving another round of gemcitabine or a gemcitabine combination instead of switching to FOLFIRINOX, which was unsuccessful in the patient and the corresponding organoids. The patient also suffered from surgical complications, ultimately preventing timely administration of chemotherapies after surgery. Patient 3′s organoids showed sensitivity to all gemcitabine-based treatments, while in clinic, the patient received a round of FOLFIRINOX before surgery followed by round of gemcitabine and gemcitabine + paclitaxel, but seemingly too late as the patient′s tumor metastasized to the liver. Given the results of the organoids, Patient 3 could have potentially benefited from receiving both gemcitabine treatments earlier in the treatment instead of FOLFIRINOX, which was ineffective for both the patient and the organoids. The late prognosis and noticeably rapid progression and short duration from diagnosis to patient′s death also makes pancreatic cancer difficult to compare with in clinic results. Ideally, a cancer with a slower progression with a more controlled, in parallel clinical trial would be needed to further confirm that temporal preclinical testing is better at predicting drug sensitives compared to continuous delivery of treatment.

A current limitation of organoid technology is that it provides an incomplete representation of the tumor microenvironment such as blood vessels, stromal components, and immune cells. Recent research has demonstrated that organoids can thrive in co-cultures with other cellular components such as patient-derived T-cells^24–26^, fibroblasts^27–29^, or even microbial and viral components^30,31^. Specifically, PDAC has been characterized by a preponderant stromal component that largely exceeds the epithelial component, and the PDAC associated fibroblasts are known to secrete factors that stimulate tumor growth, cell survival, and metastasis^1,25^. Another caveat to acknowledgement is the use of Iriontecan (CPT-11) instead of its active metabolite SN-38 that is bioactivated through hydrolyzation in the liver, the use of this compound instead would further improve the accuracy of our model. In future research, improvements could be made to our platform through the incorporation of other cellular elements such as patient-derived stromal fibroblasts or immune components to further create a more realistic tumor microenvironment. Through this incorporation, our platform could potentially accelerate investigations into how the stroma modulates disease progression, imparts resistance to drugs, and affects therapeutic response.

## Methods

### Design and fabrication of the platform

We designed and fabricated both devices based on previously developed standard microfluidic protocols^32^. In summary, both devices were designed using AutoCAD (Autodesk Inc. San Rafael, CA, USA) and processed using photolithography techniques to make silicon-based molds that were then used to cast the devices from polydimethylsiloxane (PDMS) using standard soft lithography procedures. The design of the multiplexer control device was based on previously developed techniques allowing for multiple fluidic configurations and control with a minimal number of valves^32–36^. The multiplexer control device is composed of two PDMS-based layers, a flow layer and a control layer, bonded together onto a glass slide to produce push-up valve configuration for fluidic control. The 3D culture chamber device consisted of two PDMS slabs: the channel layer for fluidic exchange and chamber layer consisting of the 200-well array. Both the channel layer and the chamber layer molds were constructed out of SU-8 3050, while the control layer and flow channels of the multiplexer control device were produced with SU-8 3025 (MicroChem, Westborough, MA, USA). Additionally, AZ-50x (AZ Electronic Materials, Luxembourg) was used to construct the valves of the multiplexer control device. The photoresists were spun to a minimal height of 450 μm for the channel layer and 600 μm for the chamber wells. The multiplexer device was spun to 75 μm for the control layer and the AZ based valves and 90 μm for the fluidic flow layer. Using standard soft lithography procedures, 72 g of PDMS (10:1 monomer/catalyst ratio) was mixed, debubbled, and poured over the trimethylchlorosilane-treated silicon mold for both the channel layer and flow layer of the multiplexer device. The thinner chamber array used 15 g of PDMS. The control layer of the multiplexer mold was spin coated at a speed of 1800 rpm. The PDMS for all molds was cured at 80 °C for at least an hour. The channel layer was punched for inlet and outlet holes when the curing process was completed. While, the flow and control layer of the multiplexer were plasma treated, aligned, and set with at least 4 h of thermal bonding before the inlet and outlet holes were punched. The multiplexer control device was then bonded to a glass slide and cured overnight at 80 °C before use. These methods produce devices with low variation and high reproducibility with less than 2% coefficient of variation (CV, *n* = 6) for the height of the chamber and channel and almost 0% (0.4, 0.002) for the width (x and y dimensions) of the wells and channels.

### Human specimens, isolation, and culture of cancer organoids

Between 2014 and 2017, tumor samples were collected from human pancreatic ductal adenocarcinoma cancer (PDAC) patients under IRB12-1108 and IRB13-1149. Samples were obtained from patients undergoing pancreatic resections at The University of Chicago Medicine (UCM) facilities. Tumor samples were digested and established into organoids according to established protocols previously published^9^. Briefly, organoids were grown and cultured by embedding dissociated tumor cells in growth-factor-reduced (GFR) Matrigel (Corning, 356231) and cultured in complete media (Intesticult [Stemcell Technologies, 6005], A83-01 [0.5 µM, Sigma, SML0788], fibroblast growth-factor 10 [FGF10, 100 ng/ml, Gibco, PHG024], Gastrin I [10 nM, Sigma, 17105-041], N-acetyl-L-cysteine [10 mM, Sigma, A9165], Nicotinamide [10 mM, Sigma, N0636], B27 supplement [1x, Gibco, 17504-044], Primocin [1 mg/ml, InvivoGen, ant-pm-1], and Y-27632 [10.5 *μ*M Tocris, 1254]). Organoids were passaged via mechanical dissociation and TrypLE Express (Fisher Scientific, 12605-010) to single cells before being loaded on to the platform.

### Cell culture and organoid loading on platform

The chamber array layer is placed on a glass slide (75 × 50 × 2 mm) and single cells or premature organoids were embedded in 70% cold Matrigel and 30% organoid growth media and manually pipetted into each individual chamber. The chamber layer with the corresponding channel layer are placed together and nested inside the microscope plate holder (Nikon), which has been drilled to specifications to accommodate the six screws that corresponded with a machine-shopped process piece of polycarbonate to apply an even pressure on the two piece PDMS layer that in return reversibly bond the device with knobs screwed down on each of the screws.

### Multiplexer control device setup

Valve control channels of the multiplexer control device were connected to miniature pneumatic solenoid valves (Festo, Switzerland) that were controlled with a custom designed MATLAB (MathWorks, USA) graphical user interface with tab-delimited text (e.g., Excel) or csv compatible file for automation instructions. Through visual confirmation and fluidic testing, optimal closing pressures for the push-up valves were typically between 35 and 40 psi. The solutions were connected to the inlets of the multiplexer and pressurized at 5 psi. Vials to collect excess waste and solutions from wash steps are also connected. The multiplexer control device is then connected to the organoid culture chip by both the inlets and outlets of the chip. If bubbles accidently arise in the channels full of media, pressurization of the media from the inlet with the outlet valve closed can remove them. Once connected, the custom MATLAB software delivers the desired fluidic supply to the organoids or other 3D cellular culture. Before the drug treatment experimental, optimal growth media exchange/feeding occurred automatically every 38 h.

### Live-cell fluorescence microscopy and environmental control

The completed two-part organoid culture platform was housed on an automated translational stage of an inverted microscope (Nikon Eclipse Ti) that is encased inside an enclosure designed for cellular environmental control (Life Imaging Service GmbH, Basel, Switzerland). The enclosure features machinery to control the temperature, humidity, and CO_2_ gas composition to maintain the cell culture^12^. The optimal parameters consist of constant 37 °C temperature, 5% CO_2_ level, a humidity flow rate of 25–30 l/h, and a relative humidity level set to 100%. The media reserves for the organoids are also kept inside the enclosure to be maintained at ample temperature. Images of the organoids in the platform during the experiment were acquired via the supplied microscope software that is capable of automatically acquiring images at different positions, *Z*-planes/stacks, and in multiple color channels (NIS-Elements software, Japan). A digital complementary metal-oxide semiconductor (CMOS) camera (ORCA-Flash 4.0, Hamamatsu, Japan) imaged the organoids using a ×10 objective at 2–4-h intervals. To monitor and measure the organoid growth over time, the acquired phase contrast images were analyzed using an automated organoid segmentation pipeline with manual curation of segmented images. For quantification of cellular death and apoptosis, the fluorescent images were analyzed using a MATLAB script that extracts the average fluorescence intensity of the desired fluorescent marker for each segmented organoid and each time-lapse image.

### Qualification of H&E architecture

Organoids were fixed with 10% formalin, paraffin embedded and sectioned (5 μm) then stained with hematoxylin and eosin (H&E) for histopathological evaluation. A gastrointestinal pathologist classified the organoids based on tumor cell appearance, differentiation, and gland structure. Previously published data on the same organoids used here (Romero Calvo et al.) showed a more valuable overall representation of tumor differentiation with H&E tumor cell appearance and gland structure than individual immunohistochemical markers.

### Culture of other 3D cellular structures and organoids

Human colonic organoids were obtained from University of Chicago′s Dr. Sonia Kupfer′s laboratory were processed and cultured on the platform similarly to PDAC organoids. To recap, similar to the PDAC organoids, the colonic organoids were spilt into single cells and embedded into Matrigel before loading onto the platform. They were then grown with growth media previously described^37^ and also reiterated here: the colonic organoid basal media contained Advanced Dulbecco′s Modified Eagle′s Medium/F12, penicillin/streptomycin, 10 mM HEPES, and 2 mM GlutaMAX (all obtained from Thermo Fisher Scientific). The basal media was supplemented with Gastrin I (10 nM, Sigma, 17105-041), N-acetyl-L-cysteine (10 mM, Sigma, A9165), recombinant mouse Noggin (100 ng/mL, PeproTech), recombinant mouse EGF (50 ng/mL, Thermo Fisher Scientific), recombinant human IGF-1 (BioLegend), B27 supplement (1×, Gibco, 17504-044), fibroblast growth-factor 2 (FGF2, 50 ng/ml, Gibco), recombinant human R-spondin 1 (1 μg/ml), A83-01 [0.5 μM, Sigma, SML0788], and 50% Afamin-Wnt-3A serum-free conditioned medium (Mihara et al.). We maintained the MDA-MB-231 human breast cancer cell line in DMEM, high glucose/pyruvate, (11995115, Gibco) containing 10% fetal bovine serum (FBS). Cells were resuspended in 1:1 Matrigel: Growth Media Solution before being seeded into the platform. Growth protocol was then carried out in a similar fashion as the organoid culture with feeding every 48 h.

### Drug screening experiments

*Real-time viability*: Relative apoptosis and viability of organoids was determined by measuring apoptosis with a caspase 3/7 fluorescence marker (Essen Bioscience 4400) and cellular death with propidium iodide fluorescence maker (Thermo Fisher P3566) simultaneously in real-time using the described microscope platform. After 1–2 weeks of growth on our platform, the organoids were treated with desired chemotherapy drugs once they reached around 100 μm diameter in size. Cells were incubated with the caspase 3/7 (5 μM) and propidium iodide (1.5 μM) along with the desired drug or drug combination. The desired drug solution was automatically delivered with the multiplexer control device according to the preset program. Imaging began 30 min after the first drug delivery to ensure proper incorporation of the fluorescent markers and continued for the complete 72-h time period every 2 or 4 h. The fluorescence time-lapse images intensities were measured, acquired with MATLAB, and normalized to the positive control (staurosporine, 10 mM). The acquired apoptosis (caspase 3/7) fluorescent intensities were analyzed using the trapezoid rule for numerical integration and averaging of the three independent experimental replicates for each subset of treatments along with the standard error of the mean of the replicates (s.e.m.). The wash and waste capabilities of the multiplex control device allowed thorough wash and cleansing of the fluidic controller between different drug treatments. Fluorescent intensity of the entire experiment for each patient and replicates were averaged and presented in drug sensitivity heatmap (*n* = 3).

All treatments were normalized to each patient′s corresponding positive control and to the first time point to prevent any possible well to well variation. To resemble traditional plate-based, SOC drugs were used to treat the tumor organoids at 4- and 72-h durations. To mimic and mirror real-life patient chemotherapy treatments, combination treatments were given temporally/sequentially as outlined in Table 1. Tumor organoid culture variation between the wells were measured based on the organoid size distribution with a coefficient of variation (CV, *n* = 6) of 12.8%, which was accounted for during the image analysis with replicates and normalization to positive control and to the first time point in each treatment group. Docetaxel (01885, Sigma); Paclitaxel (T7191, Sigma); Gemcitabine Hydrochloride (G6423, Sigma); 5-Fluorouracil (F6627, Sigma); Cisplatin (232120, Sigma); Oxaliplatin (AG-CR1-3592, AdipoGen); Irinotecan Hydrochloride (CPT-11) (I1406, Sigma); Staurosporine (NC0748115, Fisher Scientific).

### Statistics and reproducibility

Statistical analysis was performed with MATLAB and repeated measures ANOVA and one-way ANOVA was used as indicated (*p* < 0.05). All experiments were conducted with samples sizes of a minimum of three. All microscopic images provided in the figures are representative of the entire sample size and have been reproducible in independent replicates and experiments.

### Reporting summary

Further information on research design is available in the Nature Research Reporting Summary linked to this article.

## Supplementary information

Supplementary Information

Description of Additional Supplementary Files

Supplementary Movie 1

Supplementary Movie 2

Supplementary Movie 3

Supplementary Movie 4

Supplementary Movie 5

Supplementary Movie 6

Supplementary Movie 7

Supplementary Movie 8

Supplementary Movie 9

Reporting Summary

## Data Availability

The main datasets generated and analyzed are presented in the paper and/or the supplementary materials. The microfluidic chip design is available with figshare on 10.6084/m9.figshare.12424673. Any additional data related to this paper may be requested from the authors. Source data are provided with this paper.

## Source data

Source Data

## Code availability

The custom control software is available with figshare on 10.6084/m9.figshare.12424733.
