# Supplementary material for: Automated microfluidic platform for dynamic and combinatorial drug screening of tumor organoids
Source: Nat Commun. 2020 Oct 19;11:5271. doi: 10.1038/s41467-020-19058-4 (PMC7573629; doi:10.1038/s41467-020-19058-4)
Supplement: Supplementary file 1 — Supplementary Information [file 41467_2020_19058_MOESM1_ESM.pdf]

Supplementary Information

Automated Microfluidic Platform for Dynamic and Combinatorial Drug Screening of Tumor Organoids

Schuster et al.

Supplementary Movies:

Supplementary Movie 1: Continuous flow of Alexa Fluor 647 fluorescent dye, constant hold of dye with valves closed, and pulse of dye through channel to demonstrate no leakage or cross talk between the channels and the ability to control fluids to the designated channel

Supplementary Movie 2: 3D reconstruction of a human pancreatic ductal adenocarcinoma cancer (PDAC) organoids on the platform (DAPI, nucleus=blue; phalloidin, F-actin=red)

Supplementary Movie 3: Series of Z stacks taken from a group of human PDAC organoids on the platform (DAPI, nucleus=blue; phalloidin, F-actin=red)

Supplementary Movie 4: Time lapse of the growth of a normal human colonic organoid on the platform

Supplementary Movie 5: Time lapse of a breast cancer cell line, MDA-MB-231, grown into 3D aggregates on platform

Supplementary Movie 6: Time lapse of the growth of patient 1 PDAC organoids on the platform

Supplementary Movie 7: Time lapse of the growth of patient 2 PDAC organoids on the platform

Supplementary Movie 8: Time lapse of the growth of patient 3 PDAC organoids on the platform

Supplementary Movie 9: Drug montage of patient 1 PDAC organoids undergoing 72-hour drug treatments (Caspase 3/7, apoptosis = green; propidium iodide, cellular death=red)

Supplementary Figures:

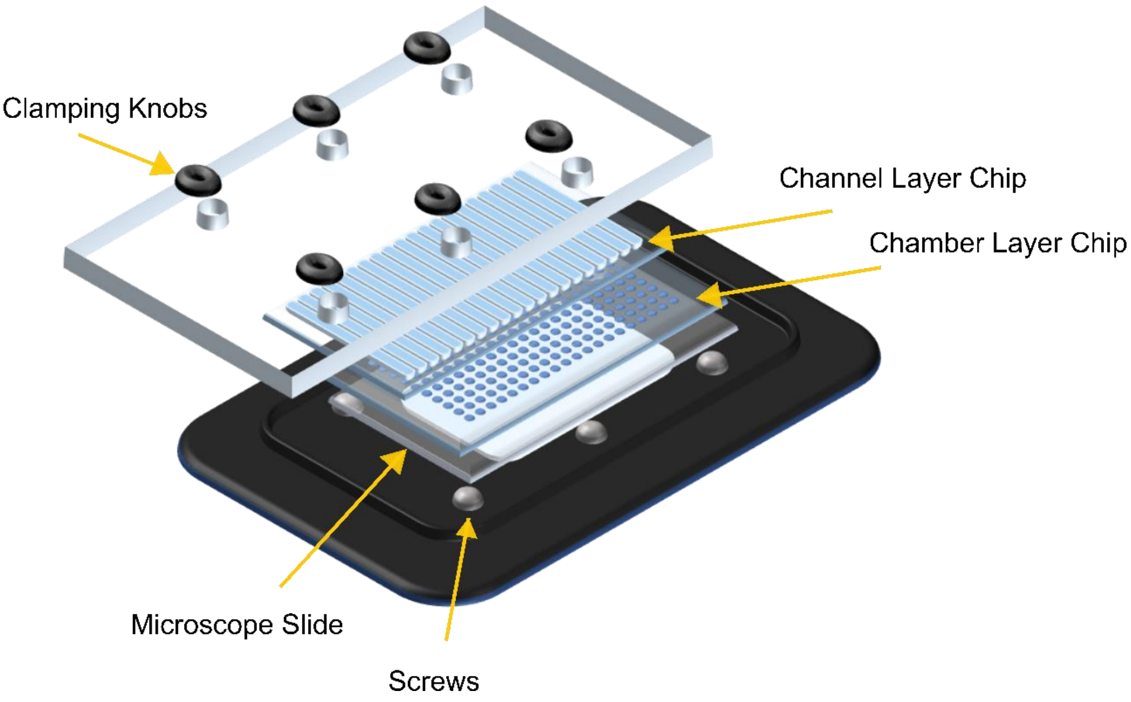

Supplementary Fig. 1. Overview of 3D Culture Chamber Platform. A microscope stage is customized with six holes for screws to provide the structure of the clamping system. A standard 50x75 mm glass slide is nested inside the screws secured to the stage. On top of the glass slide, lies the first thin layer of an air permeable silicone material (PDMS) encapsulating miniature chamber wells, 1.5 mm in diameter and 610 µM deep, which houses the 3D, gel-compatible, environment for the culture. Complementing the chamber layer, a second PDMS based channel layer goes on top for the fluidic supply to the 3D cellular culture. Finally, a clear piece of polycarbonate applies even pressure by reversibly bonding the layers with knobs screwed down to prevent any leakage or cross-contamination between the channels.

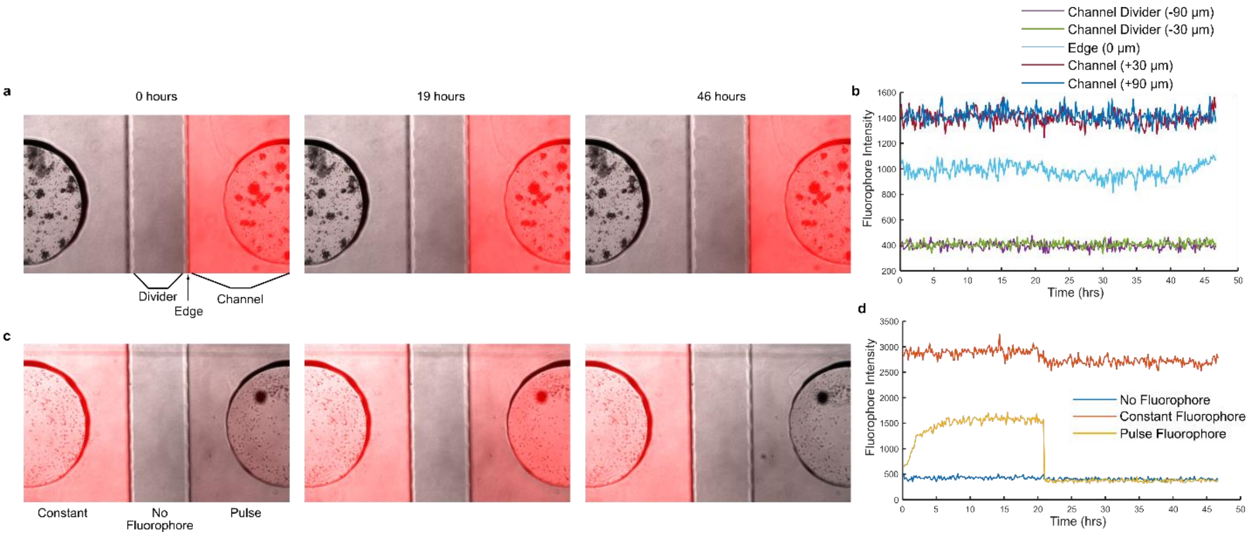

Supplementary Fig. 2. Lack of leakage, drug absorption, and cross contamination between microfluidic channels: Alexa Fluor 647 fluorescent dye (Sigma Aldrich GE25-9004) was added to specific channels and fluorescence intensity was monitored overtime to assess leakage and any cross-talk/drug contamination between channels. a-b) With the edge of a channel with the fluorophore at constant pressure as the starting point, 30 µm and 90 µm to the left of the edge in the divider region and 30 µm and 90 µm to right of the edge in the channel region was assessed for fluorescent intensity. c-d) A channel with the fluorophore, the divider between the channels (no fluorophore), and a channel that received a pulse of the fluorophore solution was measured over 48 hours. In additional to the use of fluorescent to access leakage, all drugs used in our study except paclitaxel and docetaxel are hydrophilic, and their concentrations are expected to be unchanged. For paclitaxel (and its derivative docetaxel), a study[32] found less than 5% absorption of the drug into the PDMS device. We therefore estimate that the drug concentrations inside the chambers are as expected, with no more than 5% difference than the concentrations of the stock solution.

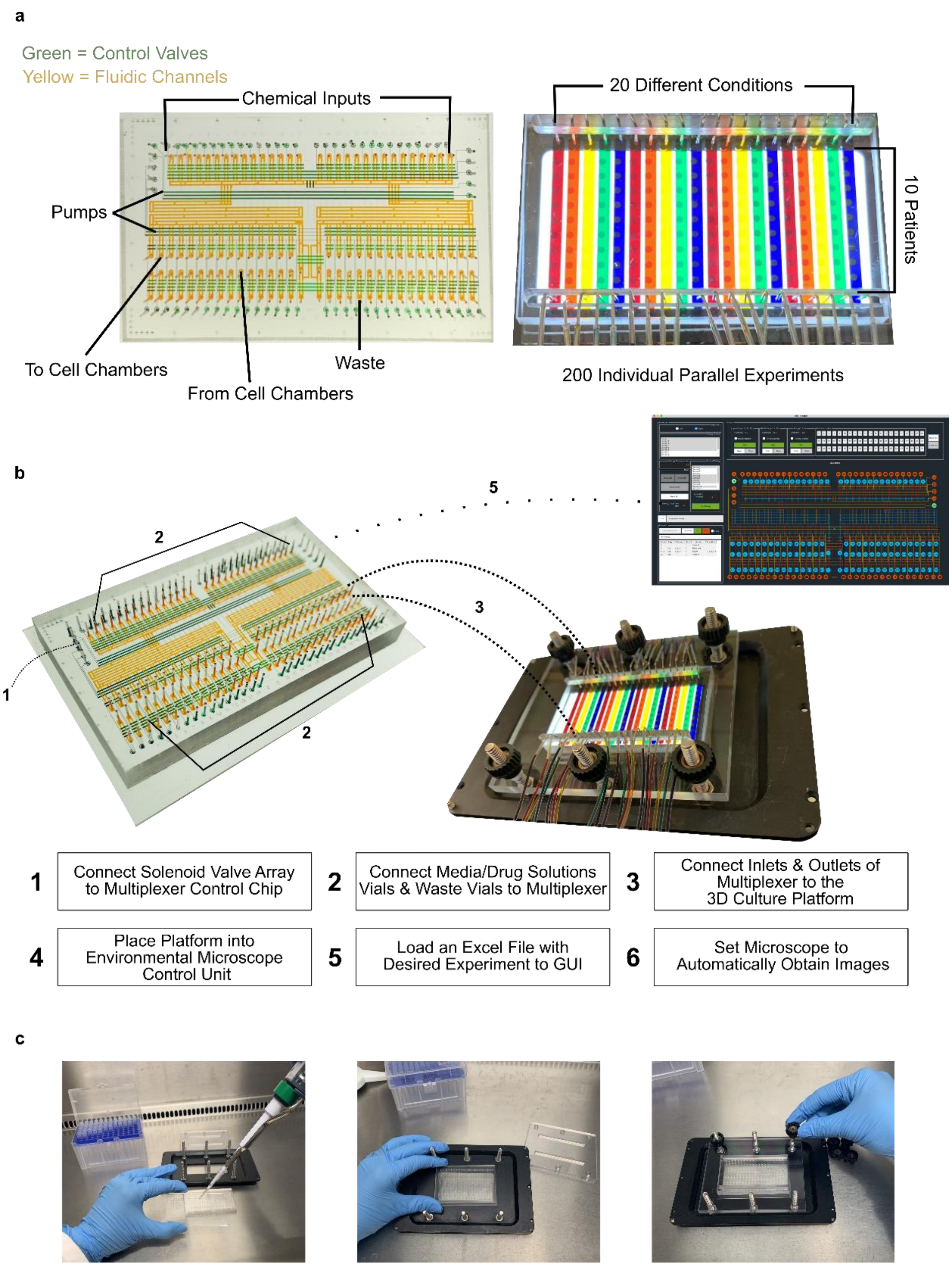

Supplementary Fig. 3. Experimental Setup. a) Features of the multiplexer control device (left) and the 3D culture chamber device (right). b) The multiplexer control chip is connected to a solenoid valve array through the outer inlets and pressurized at around 40 psi. Solutions (pressurized at 5 psi) and waste vials are connected to the appropriate inlets on the chip. The multiplexer is then connected to the culture platform before being placed onto a microscope equipped with an environmental control unit. The desired experiment is uploaded to both the solenoid valve array control computer and microscope for automatic fluidic delivery and image

acquisition. c) Organoids embedded in Matrigel or other 3D cellular culture are easily pipetted into the chamber layer, followed by assembly of the channel layer reversibly bonded with the clamping system.

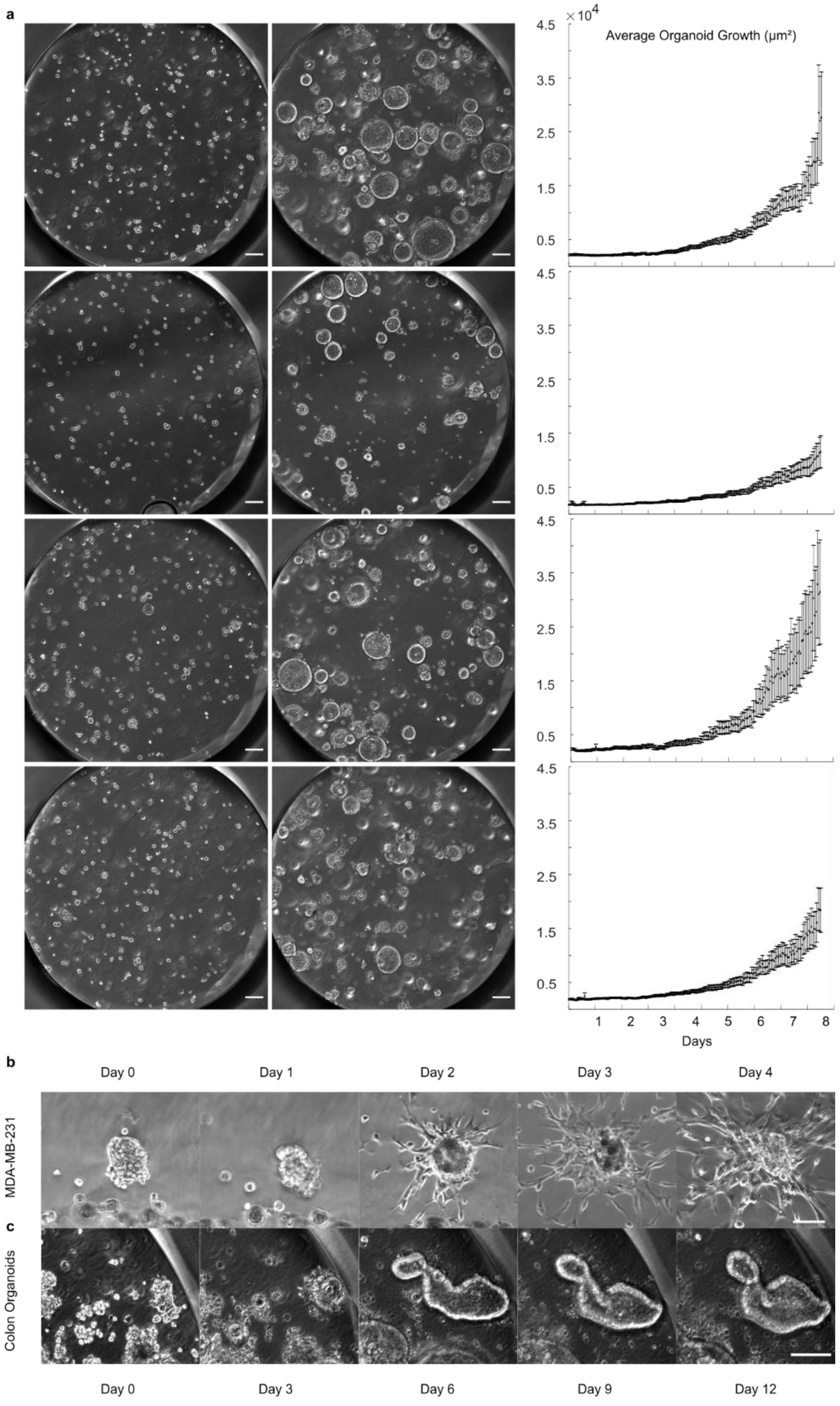

Supplementary Fig. 4. Pancreatic Cancer Organoid and Other 3D Cellular Cultures Grown on Platform. a) Examples of four different cell culture chambers growing from single cells to mature pancreatic ductal adenocarcinoma (PDAC) organoids in 8 days on the platform in parallel (scale bar *100 µm)*. b) Immortalized cancer cell lines such as MDA-MB-231, a breast cancer line, can be grown and stimulated on the platform to form cellular aggregates for drug screening or

migration studies. Other cancer organoids and non-cancer/diseased organoids such as the human normal colonic organoids shown are also compatible with this system (scale bar *100 μm)*.

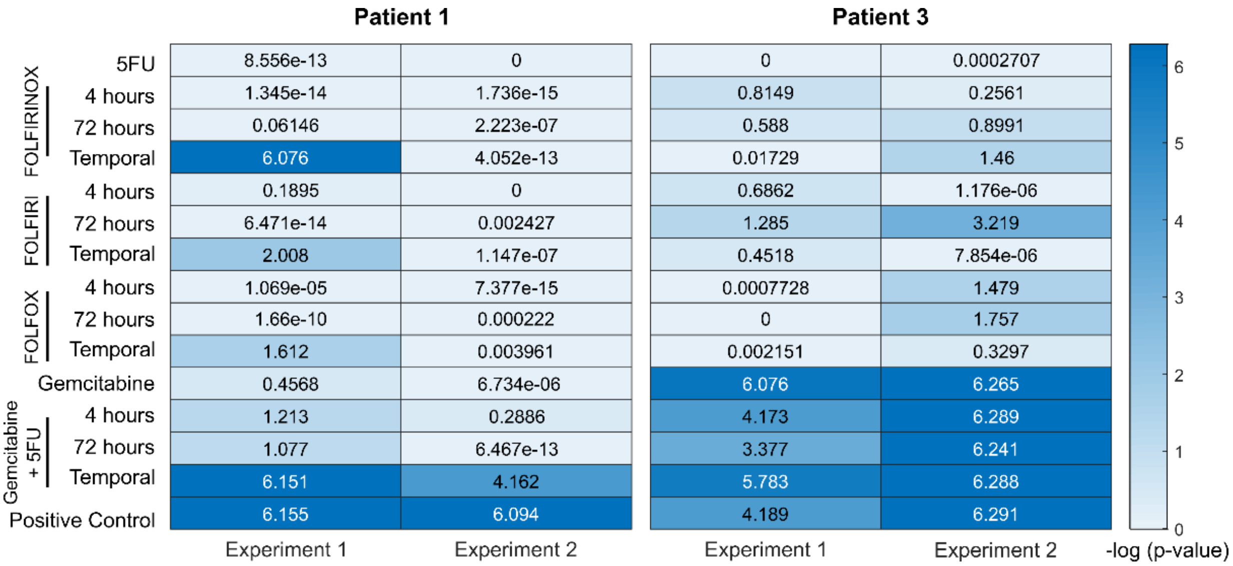

Supplementary Fig. 5. Reproducibility of the Platform. An identical experiment was repeated with two of the patients shown with a heat map of -log (p-value) of each drug treatment compared to the negative control (no drug/s). Patient 1 was repeated with varying passage numbers (Experiment 1: P41 and Experiment 2: P15), while patient 3 were both similar passages (Experiment 1: P24 and Experiment 2: P27). Overall, both patients retained the most significant and effective drug (Patient 1: gemcitabine + 5FU Temporal; Patient 2: all gemcitabine treatments; repeated measures ANOVA, n=3) for both experiments. For both experiments, the passage number did not affect the experimental results as expected[9].

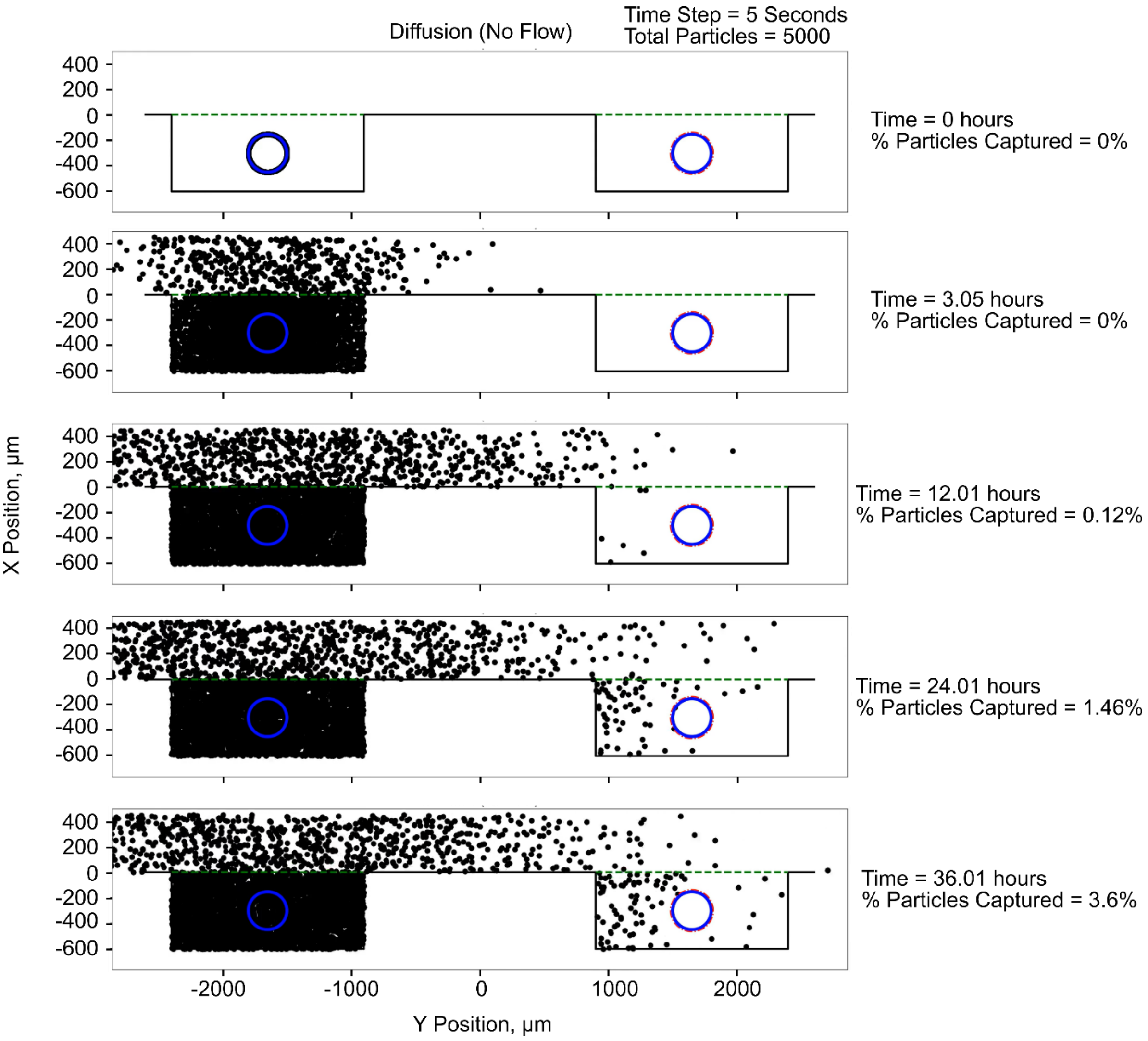

Supplementary Fig. 6. Mathematical Modeling of Potential Organoid Cross-Communication Between Microfluidic Wells. A model simulates the movement of 5,000 cytokine particles between two individual wells of organoids within one microfluidic channel. Using a time step of 5 seconds, the model simulated 36 hours of cytokine secretion and the potential capture of those particles to the neighboring well of organoids. The model demonstrated that low overall cytokine cross talk (3.6%) occurs between neighboring wells of organoids.

Supplementary Note 1:

Modeling Organoid Cytokine Cross-Talk within One Microfluidic Channel

In order to determine if an organoid culture in a single well could be influenced by cellular factors secreted by neighboring wells of organoids in the same microfluidic channel, we developed a python-based model to simulate the transfer and capture of cytokines from one organoid chamber to its neighboring chamber. The model was formulated by simulating two neighboring well chambers and the flow channel above them with the same well dimensions as the actual device. The number of cells per well were estimated to be 2,500 cells with the organoids having an average diameter of 300 μm. The program simulates the release of cytokines from the surface of the left organoid over a period of 36 hours. The cytokines will diffuse slowly via a random walk in the well until they transition above the green dotted line (representing the Matrigel barrier) and then enter the main flow channel, continue to diffuse, and cross into the gel of a neighboring well. If they go into the second well, they are considered as captured once they come in contact with the second organoid (the red dotted line), are removed from the simulation, and the capture counter is updated. This process is simulated for 36 hours as at each 36-hour cycle the feeding or drug medium is exchanged and that would remove any cytokines in the channels. Degradation or half-life of cytokine was not considered. Diffusion coefficient for the medium: 3E-10 (m^2/sec). Difussion coefficient for the matirgel: 5.15e-12 (m^2/sec). The model demonstrated that low overall cytokine cross talk (3.6%) occurs between neighboring wells of organoids. Given that cancer organoids are not composed of cells that are specialized secretors of signaling factors (i.e. immune cells), and that the overall molecular transport is very small, it is believed that the drug studies will not be influenced in a significant way by secreted cross-talk factors.
