# Supplementary material for: Automated microfluidic platform for dynamic and combinatorial drug screening of tumor organoids
Source: Nat Commun. 2020 Oct 19;11:5271. doi: 10.1038/s41467-020-19058-4 (PMC7573629; doi:10.1038/s41467-020-19058-4)
Supplement: Supplementary file 2 — Description of Additional Supplementary Files [file 41467_2020_19058_MOESM2_ESM.pdf]

Description of Additional Supplementary Files

Supplementary Movie 1: Continuous flow of Alexa Fluor 647 fluorescent dye, constant hold of dye with valves closed, and pulse of dye through channel to demonstrate no leakage or cross talk between the channels and the ability to control fluids to the designated channel

Supplementary Movie 2: 3D reconstruction of a human pancreatic ductal adenocarcinoma cancer (PDAC) organoids on the platform (DAPI, nucleus=blue; phalloidin, F-actin=red)

Supplementary Movie 3: Series of Z stacks taken from a group of human PDAC organoids on the platform (DAPI, nucleus=blue; phalloidin, F-actin=red)

Supplementary Movie 4: Time lapse of the growth of a normal human colonic organoid on the platform

Supplementary Movie 5: Time lapse of a breast cancer cell line, MDA-MB-231, grown into 3D aggregates on platform

Supplementary Movie 6: Time lapse of the growth of patient 1 PDAC organoids on the platform

Supplementary Movie 7: Time lapse of the growth of patient 2 PDAC organoids on the platform

Supplementary Movie 8: Time lapse of the growth of patient 3 PDAC organoids on the platform

Supplementary Movie 9: Drug montage of patient 1 PDAC organoids undergoing 72-hour drug treatments (Caspase 3/7, apoptosis = green; propidium iodide, cellular death=red)
