# Supplementary material for: Automated microfluidic platform for dynamic and combinatorial drug screening of tumor organoids
Source: Nat Commun. 2020 Oct 19;11:5271. doi: 10.1038/s41467-020-19058-4 (PMC7573629; doi:10.1038/s41467-020-19058-4)
Supplement: Supplementary file 12 — Reporting Summary [file 41467_2020_19058_MOESM12_ESM.pdf]

nature research

Corresponding author(s): Savas Tay

Last updated by author(s): 09/10/2020

Reporting Summary

Nature Research wishes to improve the reproducibility of the work that we publish. This form provides structure for consistency and transparency in reporting. For further information on Nature Research policies, see Authors & Referees and the Editorial Policy Checklist.

Statistics

For all statistical analyses, confirm that the following items are present in the figure legend, table legend, main text, or Methods section.

n/a	Confirmed	
☐	☒	The exact sample size (*n*) for each experimental group/condition, given as a discrete number and unit of measurement
☐	☒	A statement on whether measurements were taken from distinct samples or whether the same sample was measured repeatedly
☐	☒	The statistical test(s) used AND whether they are one- or two-sided<br>*Only common tests should be described solely by name; describe more complex techniques in the Methods section.*
☒	☐	A description of all covariates tested
☐	☒	A description of any assumptions or corrections, such as tests of normality and adjustment for multiple comparisons
☐	☒	A full description of the statistical parameters including central tendency (e.g. means) or other basic estimates (e.g. regression coefficient) AND variation (e.g. standard deviation) or associated estimates of uncertainty (e.g. confidence intervals)
☐	☒	For null hypothesis testing, the test statistic (e.g. *F*, *t*, *r*) with confidence intervals, effect sizes, degrees of freedom and *P* value noted<br>*Give P values as exact values whenever suitable.*
☒	☐	For Bayesian analysis, information on the choice of priors and Markov chain Monte Carlo settings
☒	☐	For hierarchical and complex designs, identification of the appropriate level for tests and full reporting of outcomes
☒	☐	Estimates of effect sizes (e.g. Cohen's *d*, Pearson's *r*), indicating how they were calculated

Our web collection on statistics for biologists contains articles on many of the points above.

Software and code

Policy information about availability of computer code

Data collection	Microscopy data collection was obtained with NIS-Elements AR 5.20.00 from Nikon, the customized control software was developed using MATLAB R2017 (10.6084/m9.figshare.12424733) with Microsoft Excel 2016 to write the .csv files, AutoCAD 2016 was used for device design
Data analysis	Data was analyzed and plots were created as described in the methods section using MATLAB R2017

For manuscripts utilizing custom algorithms or software that are central to the research but not yet described in published literature, software must be made available to editors/reviewers. We strongly encourage code deposition in a community repository (e.g. GitHub). See the Nature Research guidelines for submitting code & software for further information.

Data

Policy information about availability of data

All manuscripts must include a data availability statement. This statement should provide the following information, where applicable:

- Accession codes, unique identifiers, or web links for publicly available datasets
- A list of figures that have associated raw data
- A description of any restrictions on data availability

The main datasets generated and analyzed are presented in the paper and/or the supplementary materials. Source data for all numerical figures are provided with the paper. The microfluidic chip design is available with figshare on 10.6084/m9.figshare.12424673. Any additional data related to this paper may be requested from the authors.

Field-specific reporting

Please select the one below that is the best fit for your research. If you are not sure, read the appropriate sections before making your selection.

☒ Life sciences ☐ Behavioural & social sciences ☐ Ecological, evolutionary & environmental sciences

For a reference copy of the document with all sections, see nature.com/documents/nr-reporting-summary-flat.pdf

Life sciences study design

All studies must disclose on these points even when the disclosure is negative.

Sample size	Each patient had 3 replicate samples for each treatment group, no sample size calculation was performed.
Data exclusions	No data were excluded from the analyses.
Replication	The entire experiment for two patients was repeated a second time to confirm reproducibility, Each experiment had at least 3 replicates from independent assay units on the same device. All attempts at reproducibility was successful.
Randomization	All measurements recorded, no selection required.
Blinding	Blinding was not relevant as all of the image data was analyzed through a matlab based program with no bias.

Reporting for specific materials, systems and methods

We require information from authors about some types of materials, experimental systems and methods used in many studies. Here, indicate whether each material, system or method listed is relevant to your study. If you are not sure if a list item applies to your research, read the appropriate section before selecting a response.

Materials & experimental systems

n/a	Involved in the study	
☒	☐	Antibodies
☐	☒	Eukaryotic cell lines
☒	☐	Palaeontology
☒	☐	Animals and other organisms
☐	☒	Human research participants
☒	☐	Clinical data

Methods

n/a	Involved in the study	
☒	☐	ChIP-seq
☒	☐	Flow cytometry
☒	☐	MRI-based neuroimaging

Eukaryotic cell lines

Policy information about cell lines

Cell line source(s)	ATCC MDAMB231
Authentication	Cell line identity was not authenticated.
Mycoplasma contamination	All cell lines tested negative for mycoplasma contamination
Commonly misidentified lines (See ICLAC register)	No commonly misidentified cell lines were used in the study.

Human research participants

Policy information about studies involving human research participants

Population characteristics	All patient data was de-identified.
Recruitment	Patients were recruited according to the clinical trial protocols: IRB12-1108 and IRB13-1149
Ethics oversight	IRB

Note that full information on the approval of the study protocol must also be provided in the manuscript.
